# Penile Calciphylaxis: A Rare-Case Report of Calcific Uremic Arteriolopathy in a Patient with End-Stage Renal Disease and Severe Vascular Insufficiency

**DOI:** 10.7759/cureus.70377

**Published:** 2024-09-28

**Authors:** Laura Chagam, Prince Patel, Sara El Aryan, Michael W Fountain

**Affiliations:** 1 Medicine, Lake Erie College of Osteopathic Medicine, Bradenton, USA; 2 Medicine, Rowan-Virtua School of Osteopathic Medicine, Stratford, USA; 3 Urology, AdventHealth Waterman, Tavares, USA

**Keywords:** acute gangrenous cholecystitis, end stage renal disease (esrd), penile calciphylaxis, severe sepsis, supra-therapeutic inr

## Abstract

Penile calciphylaxis is a rare and life-threatening condition, most commonly seen in patients with end-stage renal disease (ESRD) undergoing dialysis. The pathophysiology includes calcification of small blood vessels, leading to ischemic changes, most commonly affecting the extremities. Treatment modalities vary based on the history and condition of the patient. In this report, we present a case of a 58-year-old male with ESRD on hemodialysis (HD) who presented with sloughed necrosis of his penis due to systemic calciphylaxis. Despite the severity of the necrosis, he was deemed unfit to undergo surgical intervention due to his complex comorbidities and was instead treated with less invasive local wound care.

## Introduction

Penile calciphylaxis, also known as calcific uremic arteriopathy (CUA) of the penis, is a rare and life-threatening sequela of end-stage renal disease (ESRD), especially for those undergoing dialysis [1]. Penile CUA has a poor prognosis with an overall mortality rate of 48% [2]. The incidence of penile calciphylaxis is reported to be approximately 6%, predominantly occurring in diabetic patients with chronic kidney disease (CKD) and those with high serum levels of calcium and phosphate [3]. 

CUA is characterized by the calcification of small vessels, thought to be caused by impaired inhibition of calcium phosphate precipitation [4]. This condition typically affects areas with high adiposity, most commonly the trunk and upper and lower extremities. It has also been documented that one of the highest risk factors for the development of CUA is diabetes mellitus [5]. 

There are no evidence-based guidelines for the treatment of penile CUA, but therapeutic options include wound care, parathyroidectomy, penectomy, and revascularization [1,5]. Additionally, it has been proposed that sodium thiosulfate could be used as an adjunctive therapy due to its potential chelating properties and vasodilatory effects, however clinical trials have not yet confirmed its efficacy and safety [6,7]. 

## Case presentation

A 58-year-old Hispanic male presented to the emergency department with lower extremity swelling, penile discharge, and scrotal pain for several days. He had a past medical history of ESRD on hemodialysis (HD) for over 10 years, renal cell carcinoma status post left nephrectomy in 2013, coronary artery disease status post coronary artery bypass graft in 2018, mitral and tricuspid valve repair, atrial fibrillation on warfarin therapy, chronic obstructive pulmonary disease (COPD) on 2 l of oxygen at home, hypertension, pulmonary hypertension, and heart failure with preserved ejection fraction (HFpEF). His home medications included cinacalcet, vitamin D2, atorvastatin, midodrine, budesonide/formoterol inhaler, and albuterol inhaler. He had a recent history of multiple hospital admissions including hypoxic respiratory failure secondary to pulmonary edema, septic shock from *Klebsiella* bacteremia, *Escherichia coli *pneumonia, and acute on chronic renal failure. He initially denied any purulent penile discharge and was able to produce a small amount of urine with dysuria. He denied any fevers, chills, chest pain, abdominal pain, nausea, vomiting, or diarrhea.

**Figure 1:**
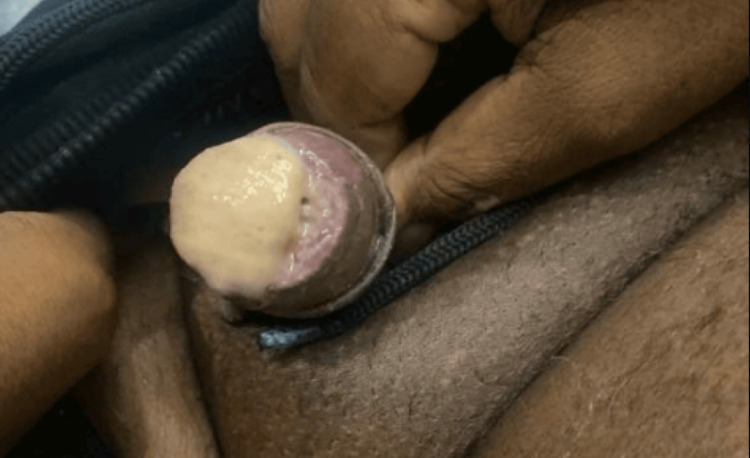
Sloughed necrosis of glans penis

**Figure 2:**
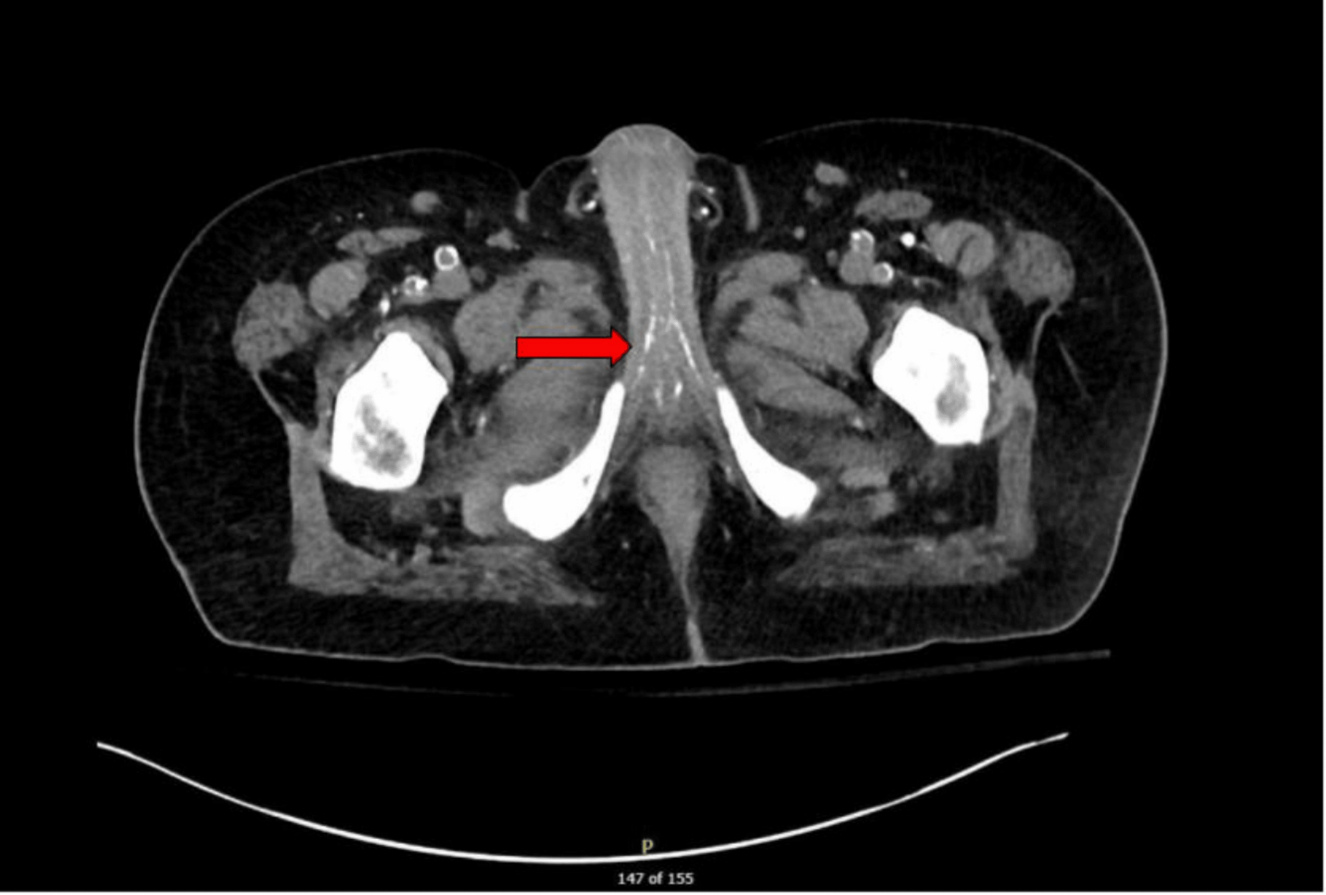
CT abdomen pelvis without IV contrast Axial image delineating extensive calcification of the cavernosal arteries (red arrow)

**Figure 3:**
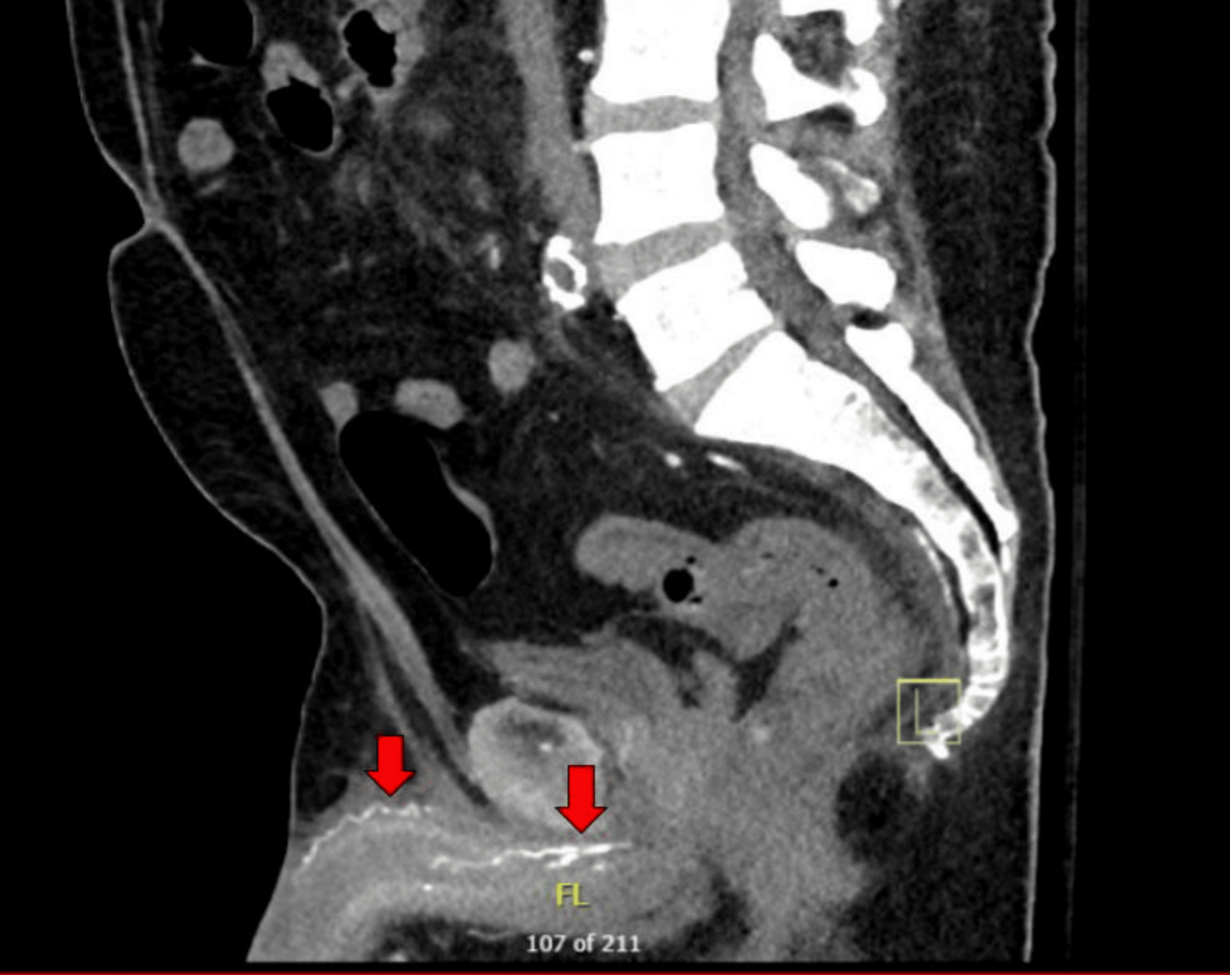
CT abdomen pelvis without IV contrast Sagittal plane delineating extensive calcification in the cavernosal arteries (red arrows)

**Figure 4:**
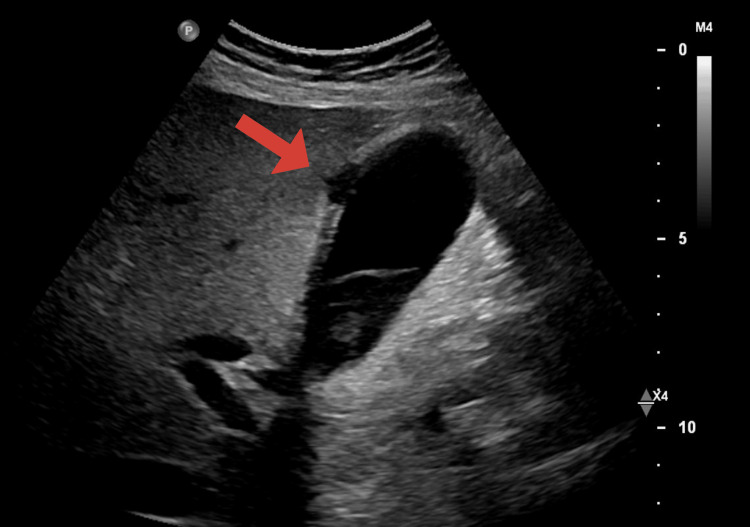
Gallbladder ultrasound Findings were concerning for acute gangrenous cholecystitis with focal intrahepatic perforation along the gallbladder fossa of the liver. There was increased conspicuity of the pericholecystic fat.

Initial vital signs on presentation to the emergency department were stable, except tachycardia at a rate of 111 beats per minute and 100% oxygenation on 2 l of oxygen by nasal cannula. Laboratory tests included a complete blood count with a comprehensive metabolic panel. The test results included leukocytosis, baseline hemoglobin of 8.9 g/dL, mild hyponatremia, elevated alkaline phosphatase, elevated lactate levels, and CKD with a glomerular filtration rate (GFR) of 5.5 mL/min. These findings met the criteria for systemic inflammatory response syndrome (SIRS). His calcium levels remained low to normal, ranging from 7 to 8.5 mg/dL, phosphorus was within normal limits at 3.10 mg/dL, and parathyroid hormone (PTH) was within normal limits (Table 1). Physical exam revealed bilateral lower extremity edema with the presence of several crusted lesions and sloughed necrosis of glans penis (Figure 1). CT scan of the abdomen and pelvis revealed right perinephric and periureteral stranding, hepatic steatosis, gallbladder wall thickening without gallstones, and pulmonary edema. It also showed diffuse calcification of the cavernosal arteries of the penis, calcified pleural plaques, renal basilar calcifications, and diffuse idiopathic skeletal hyperostosis (Figures 2, 3). Gallbladder ultrasound revealed acute gangrenous cholecystitis with focal intrahepatic perforation, wall thickness of 10 mm, and multiple rim calcified renal cysts (Figure 4).

**Table 1:** Laboratory values All other values are within normal limits unless stated otherwise. WBC: White blood cell count; GFR: Glomerular filtration rate; PTH: Parathyroid hormone

	Reference Range	Laboratory Value
WBC	3.30 - 10.60 × 10^3^/uL	25.28
Hemoglobin	14.0 - 18.0 g/dL	8.9
Sodium	136 - 145 mmol/L	135
Potassium	3.5 - 5.1 mmol/L	4.4
Alkaline Phosphatase	38 - 126 U/L	279
Lactic Acid	0.5 - 2.20 mmol/L	4.80
Creatinine	0.40 - 1.4 mg/dL	4.40
GFR	>60 mL/min	5.5
Calcium	8.0 - 10.2 mg/dL	8.2
Phosphorous	2.40 - 4.70 mg/dL	3.10
PTH	15.00 - 65.00 pg/mL	42.88

He was admitted to the hospital and received IV piperacillin/tazobactam 4.5 g every six hours and continuous 5% dextrose in lactated Ringer's solution (D5LR). Given the complexity of the patient's presentation, he was managed by a multidisciplinary team that included a general surgeon, nephrologist, and urologist.

Based on clinical, laboratory, and radiologic findings, the diagnoses of acute septic gangrenous cholecystitis and penile and lower extremity calciphylaxis were made. On day two of admission, he went into septic shock with a blood pressure of 81/38, which was multifactorial, and was transferred to the intensive care unit, where he received vasopressors. His blood cultures revealed *Escherichia coli *bacteremia resistant to ciprofloxacin. and he was given IV meropenem. Repeat laboratory findings revealed supratherapeutic international normalized ratio (INR) of 10.4, stabilizing after vitamin K, prothrombin complex concentrate, and four units of fresh frozen plasma. However, his coagulopathy, combined with his overall comorbid status, made him a poor surgical candidate. Therefore, an interventional radiologist (IR) performed a fluoroscopic-guided percutaneous cholecystostomy catheter placement with cholangiogram for his acute gangrenous cholecystitis with perforation. The patient continued his HD treatments in the hospital three times weekly for volume overload. Due to a drop in hemoglobin to 6.8 g/L, he received one unit of packed red blood cells and epoetin alfa. After stabilization of his vital signs and reversal of coagulopathy with INR dropping to 1.88, he was downgraded to progressive care unit on day three of admission to continue supportive care. He received daily local wound care to his lower extremities and penile soft tissue necrosis, which included irrigation with sterile saline and dressing changes. He also received physical, occupational, speech, and swallow therapy for his metabolic encephalopathy. Due to lack of authorization from insurance, he was unable to be discharged to a skilled nursing facility or rehabilitation center. He was discharged home on day 20 and advised to reassess his cholecystostomy tube with general surgery after one month. He continued two weeks of IV ceftriaxone, physical therapy, HD three times a week, and local wound care to his lower extremities and penis as outpatient therapy. 

## Discussion

Penile calciphylaxis is an uncommon and multifactorial manifestation of systemic calciphylaxis. From previous studies, the established risk factors include end-stage renal failure, dialysis treatment, diabetes, hypercalcemia, hyperphosphatemia, calcium use, vitamin D use, and warfarin therapy [8,9]. In a systematic review of reported penile calciphylaxis cases conducted in 2023, 80.2% of the patients had diabetes, 78.9% of the patients received HD, 10.9% received peritoneal dialysis, and 7.6% were patients with CKD who were not receiving dialysis [2]. Our patient stood at a significant risk for calciphylaxis, given his long standing ESRD on HD, use of vitamin D, and warfarin therapy post multi-valve repair. Additionally, he had lower extremity ulcerations and a penile ulcer with sloughed necrosis, indicating the presence of systemic calciphylaxis, affecting small and medium vessels. 

Diagnosis of penile calciphylaxis can be made with a combination of clinical presentation, findings on imaging, laboratory studies, and biopsy results [2]. We found the patient’s clinical presentation sufficient to make the diagnosis of calciphylaxis and ultimately decided against the need for a biopsy. The risks of progressive necrosis, secondary infection, and poor wound healing were considered. Given our patient’s compromised state with multi-organ dysfunction, the risks were determined to outweigh the benefits of any invasive procedure, including biopsy and surgical debridement. CT scan of the abdomen and pelvis with contrast is the most sensitive imaging modality to illustrate the extent of tissue calcification, ischemia, necrotizing infection, and the presence of air [10]. However, contrast was avoided in our patient, as it typically is in this patient population, considering his long-standing renal failure. MRI can be used as a supplemental tool to visualize the extent of tissue ischemia without the use of contrast, however we did not deem it necessary given his CT findings. 

Systemic calciphylaxis involves the deposition of calcium in the tunica media layer of small and medium arteries, arterioles, and capillaries. This mechanism is distinct from that of atherosclerosis, which involves luminal narrowing of the medium and larger arteries of the body. This explains the high prevalence of manifestations in areas such as the pelvis, penis in particular, subcutaneous tissue, and distal extremities, which contain an abundant supply of small vessels [8,10]. While the pathophysiology is complex and not yet fully understood, a central mechanism seems to be a dysregulation of calcium and phosphate metabolism in patients with CKD. Elevated calcium-phosphate product precipitates in soft tissue and artery media, leading to subsequent ischemia and necrosis [11,12]. Unfortunately, prognosis of patients with penile calciphylaxis is poor. Wipattanakitcharoen et al., in 2023, have reported a mortality rate of 48%, with a median time to death of three months [2]. 

Management of penile calciphylaxis involves the use of various treatment modalities to address multifactorial concerns. Sodium thiosulfate, hyperbaric oxygen, penectomy, parathyroidectomy, conservative management, surgical debridement, vasodilatory agents, and revascularization surgery have all been described in its treatment and management [2,8,9]. Sodium thiosulfate has been shown to be effective and the proposed mechanisms involve the chelation of calcium into a soluble form, dissolvement of calcium-phosphate precipitations, vasodilatory effects, and antioxidative properties [2,11]. Additionally, parathyroidectomy was not considered due to his normal PTH levels during his visit. Per urology recommendation, our patient was not a candidate for surgical intervention given his long-standing multi-system dysfunction, septic cholecystitis, vasculopathy, coagulopathy, and anticipated complications from impaired healing potential. Therefore, our patient was solely treated with continued local wound care for his penile lesion. 

## Conclusions

This case showcases an uncommon presentation of penile necrosis from calciphylaxis in a patient with ESRD undergoing HD. The patient’s multiple comorbidities, vasculopathy, severe coagulopathy from warfarin therapy, and acute gallbladder infection compromised wound healing. Consequently, less invasive or non-surgical approaches were preferred for managing his calciphylaxis. Unfortunately, there is inadequate imaging of the wound care to the penis upon discharge, limiting detailed visual assessment of recovery. A comprehensive approach involving medical interventions tailored to the individual’s history and complications is imperative. While treatment options exist, no curative treatment for calciphylaxis is available. Timely detection and intervention by a multidisciplinary team are crucial to preventing adverse outcomes. Further research is needed to explore the underlying mechanisms and contributory factors of penile calciphylaxis. There is a need for standardization of treatment to improve outcome and prevent complications.
